# First vaccine approval under the FDA Animal Rule

**DOI:** 10.1038/npjvaccines.2016.13

**Published:** 2016-08-25

**Authors:** David W C Beasley, Trevor L Brasel, Jason E Comer

**Affiliations:** 1 Office of Regulated Nonclinical Studies, University of Texas Medical Branch, Galveston, TX, USA; 2 Department of Microbiology and Immunology, University of Texas Medical Branch, Galveston, TX, USA; 3 Sealy Center for Vaccine Development, University of Texas Medical Branch, Galveston, TX, USA; 4 Galveston National Laboratory, University of Texas Medical Branch, Galveston, TX, USA; 5 World Health Organization Collaborating Center for Vaccine Research, Evaluation and Training on Emerging Infectious Diseases, University of Texas Medical Branch, Galveston, TX, USA

## Abstract

The US Food and Drug Administration’s Animal Rule was established to facilitate licensure of new products for life-threatening conditions when traditional efficacy trials in humans are unethical or impractical. In November, 2015 BioThrax became the first vaccine to receive approval for a new indication via this pathway. The basis for this approval and use of Animal Rule or other non-traditional approval pathways for licensure of vaccines for serious conditions are discussed.

On 23 November 2015, BioThrax (Anthrax Vaccine Adsorbed) became the first vaccine to receive approval for a new indication based on the US Food and Drug Administration’s (FDA) Animal Rule.^1,2^ BioThrax was originally licensed in the 1970s and is the only vaccine approved in the United States for human use in prevention of inhalational anthrax. The newly approved indication is for the use of BioThrax in post-exposure prophylaxis (PEP) following suspected or confirmed *Bacillus anthracis* exposure, in conjunction with recommended antibiotic treatment. This followed previous Animal Rule approvals of other therapeutic or PEP compounds for inhalational anthrax, namely Raxibacumab and Anthrasil.

The Animal Rule (21 CFR 601 Subpart H for biological products, 21 CFR 314 Subpart I for drugs) was implemented to facilitate licensure of medical countermeasures for ‘lethal or permanently disabling’ conditions for which traditional efficacy testing in clinical trials involving exposure to the disease causing agent are unethical or logistically impractical, a situation which clearly applies to post-exposure treatment of inhalational anthrax. The FDA also considers the Animal Rule a pathway of last resort, when approval under any other mechanism is not possible.^3^ The regulations allow for approval of a product based on ‘adequate and well controlled’ animal studies that suggest it is ‘reasonably likely to produce clinical benefit in humans’ and require that is has been shown to have an acceptable safety profile from clinical studies in humans. Importantly, as defined in 21 CFR 601 Part H section 601.91, 4 key criteria must be met to provide evidence for effectiveness:

1. There is a reasonably well-understood pathophysiological mechanism of the toxicity of the substance and its prevention or substantial reduction by the product;
2. The effect is demonstrated in more than one animal species expected to react with a response predictive for humans, unless the effect is demonstrated in a single animal species that represents a sufficiently well-characterized animal model for predicting the response in humans;
3. The animal study endpoint is clearly related to the desired benefit in humans, generally the enhancement of survival or prevention of major morbidity; and
4. The data or information on the kinetics and pharmacodynamics of the product or other relevant data or information, in animals and humans, allows selection of an effective dose in humans.

In Canada, the National Regulatory Authority, Health Canada, can approve vaccines and other products under regulations for Extraordinary Use New Drugs, which have many similarities to the FDA Animal Rule.^4,5^ Although the European Medicines Agency (EMA) does not have an animal rule equivalent, a recent perspective from the EMA stated that this does not preclude ‘the possibility that animal models data in principle could have a critical role in the assessment’ for approval of a product via their conditional marketing authorization or marketing authorization under exceptional circumstances pathways.^6^

Products approved under the Animal Rule are subject to requirements for post-licensure studies to demonstrate clinical efficacy, when such studies become feasible; restrictions to ensure safe use of the product; and requirements for labeling to clearly indicate that approval of the product was based on efficacy studies in animals. For developers pursuing licensure under the Animal Rule, the FDA emphasizes the importance of ‘early and ongoing communications’ with the agency regarding the applicability of this pathway, suitability of proposed animal models and design of studies to demonstrate product efficacy.^7^ Importantly, for anthrax, a strong scientific consensus exists regarding the pathogenic mechanisms of inhalational anthrax infection, the protective capacity of immune responses targeting the anthrax protective antigen (PA), and the suitability of rabbit and non-human primate (NHP) models of infection to evaluate medical countermeasures against potentially lethal disease following aerosol exposure to *B. anthracis* spores. A key aspect of the process for anthrax vaccine approval via the Animal Rule was bridging of animal and human data to establish the effective dose for humans, which was defined by the FDA Center for Biologics Evaluation and Research as requiring that the ‘vaccine dose in humans elicit an immune response comparable to that of animals protected by the vaccine’.^8^ Suitable animal study designs to provide this bridging were discussed and evaluated by the FDA’s Vaccines and Related Biological Products Advisory Committee (VRBPAC) in November, 2010 providing an effective path forward for vaccine licensure.^9^ General use prophylaxis (GUP), PEP and passive immunization studies were all considered as useful to obtain information regarding efficacy of PA-based vaccines. The GUP model was recommended as the primary basis for evaluation under the Animal Rule, in large part due to challenges identified in developing a reproducible, partially protective antibiotic dosing regimen to facilitate a PEP model in NHPs and the difficulties anticipated for assessing immune responses following vaccination after anthrax challenge.^8,10^

Significantly, for development under the Animal Rule the immune markers selected for bridging studies are not expected to be robust correlates or surrogates of protection, or approval via other mechanisms would be possible.^11^ However, a key factor in bridging immunological data from animal efficacy studies to humans is the use of robust validated or well qualified assays. In order to bridge animal data to humans and estimate effectiveness of BioThrax in a PEP indication, protective antibody levels, as measured using a validated anthrax toxin neutralizing antibody (TNA) assay, were used as a basis to assess whether vaccine administration would, with reasonable certainty, protect against disease resulting from residual spores that remained after completion of the recommended course of antimicrobial therapy,^10,12^ which is typically a 60-day course of antibiotic such as ciprofloxacin.

Two gender-balanced pivotal GUP animal studies were conducted in rabbits and NHPs to determine the protective efficacy of TNA following BioThrax vaccination.^10^ Rabbits were vaccinated intramuscularly on days 0 and 28 with multiple dilutions of BioThrax (1:4, 1:16, 1:64 and 1:256). A fifth group was injected with placebo and served as a challenge control. On day 70, the animals were challenged with an aerosolized aqueous suspension of *B. anthracis* spores equivalent to 200 50% lethal doses (LD_50_). The NHP study mirrored the rabbit work but included an additional group that received 0.5 ml of undiluted vaccine. The lowest dilution used in each study protected 100% of animals and there was a clear dose response in the other groups. A pre-exposure TNA 50% neutralization factor (NF_50_) of 0.56 in vaccinated rabbits (measured at day 69) or 0.29 in NHPs (at day 70) corresponded to 70% probability of survival, the level considered by the FDA to predict a reasonable survival benefit. A single, supportive, non-pivotal, limited, non-GLP toxicology study was conducted to determine the safety and immunogenicity of BioThrax when given in conjunction with a second compound, whose name is redacted from the FDA’s published Summary Basis for Regulatory Action^10^ but was most probably human anthrax immunoglobulin (AIGIV).^13^ This compound attenuated the endogenous immune response to the vaccine. No significant toxicological findings were reported in this study. Data from proof of concept PEP animal studies were also submitted that suggested BioThrax vaccination provides added benefit over antimicrobials alone in the PEP indication.

Although the Animal Rule does not require compliance with the 21 CFR 58 regulations for Good Laboratory Practice for Nonclinical Laboratory Studies (GLP), FDA expects that pivotal studies for model definition, efficacy evaluation and that support determination of dose regimen for humans comply with GLP ‘to the extent practicable’.^7^ The pivotal NHP efficacy study with BioThrax was not performed as a GLP compliant study but was reviewed by FDA, including a testing facility inspection, to assess whether deviations from GLP adversely affected the results of the study.^10^

The potential protective capacity of the vaccine in humans was ultimately determined by assessing the proportion of clinical study subjects that achieved a TNA NF_50_⩾0.56, which corresponded to 70% probability of survival in the animal models.^10^ Three clinical studies were included in the analysis. Two studies exclusively evaluated the immunogenicity of BioThrax while the third evaluated the potential interference of BioThrax on the pharmacokinetics (PK) of ciprofloxacin (500 mg administered twice daily on days 1–6, 19–21, 33–35, 40–48) and, conversely, the effect of ciprofloxacin on the immune response to BioThrax when given using the PEP schedule and a subcutaneous dosing route. The purpose of the first study was to determine timing and peak of protective antibody response in healthy 18- to 65-year-old adults following immunization with three doses of BioThrax, at days 0, 14 and 28. Peak responses were observed on day 42, with geometric mean TNA NF_50_ of 1.672, and all subjects had seroconverted by that timepoint. Using the same vaccination schedule, protective antibody levels were further evaluated in a second study, whose primary endpoint was the proportion of subjects with TNA NF_50_⩾0.56 at day 63. Endpoints for both studies were successfully met, no significant adverse safety signals were identified and these data were ultimately used to support licensure for the PEP indication under the Animal Rule. With respect to the third study, interference of BioThrax on the PK of ciprofloxacin was not shown. Similarly, ciprofloxacin was shown not to adversely affect the immunogenicity of BioThrax when dosed using the PEP schedule. FDA concluded that data from the animal and clinical studies supported the safety and effectiveness of BioThrax for the PEP indication in healthy adults 18–65 years of age.^10^

During 2014–2015, in response to the unprecedented outbreak in West Africa, considerable effort was expended on the development and testing of candidate vaccines for Ebola virus. Several of those vaccines had been in nonclinical development during the previous decade, with an expectation that their eventual licensure would require use of the Animal Rule. After a rapid ramp-up involving international collaboration and coordination, clinical trials of two candidate vaccines and of several therapeutics were initiated in the affected West African countries beginning in late 2014.^14^ However, due to these trials occurring at the tail end of the outbreak, collection of efficacy data sufficient to support traditional licensure of any products was limited, which has prompted further discussions regarding the path to licensure for filovirus vaccines. A May 2015 meeting of the FDA VRBPAC identified three potential pathways: approval via traditional clinical efficacy, which currently seems unlikely in the absence of further large outbreaks of Ebola disease; Accelerated Approval; or the Animal Rule.^15,16^ The Accelerated Approval pathway (defined in 21 CFR 601 subpart E for biologics; 21 CFR 314 subpart H for drugs) is one of four programs established by the FDA to ‘ensure that therapies for serious conditions are approved…as soon as it can be concluded that [their] benefits justify their risks’.^17^ Accelerated Approval allows for licensure based on a surrogate or clinical intermediate endpoint that is ‘reasonably likely to predict’ clinical benefit. For Ebola vaccines, this surrogate endpoint may be levels of total or neutralizing antibodies and would need to be defined on the basis of immunological data from animal studies, and naturally infected or exposed/protected humans, including data from the West African clinical trials.^11^ Like the Animal Rule, approvals under this mechanism are expected to involve considerable discussion with the FDA during development, require clinical demonstration of safety, and must be followed by post-licensure studies to demonstrate clinical efficacy.^17^ Both Animal Rule and Accelerated Approval mechanisms can be expected to require combinations of data from clinical and animal studies to meet the ‘reasonably likely’ benchmarks for predicting protection of humans against disease. A significant question for application of these two pathways is whether the combined animal and clinical data that are now available for Ebola are sufficient to identify an immunological or other endpoint that can serve as an acceptable surrogate in regulatory agency decision making for predicting protective efficacy in humans.

Although the recent approval of BioThrax for PEP of inhalational anthrax does not represent approval of an entirely novel vaccine via the Animal Rule, it is a significant step forward in the application of this regulation to the approval of new vaccines and other medical countermeasures. Ongoing evaluation of candidate Ebola vaccines will provide further clarification of the FDA’s expectations for consideration of these products under the Animal Rule or Accelerated Approval pathways, and will provide opportunities to evaluate specific requirements for clinical and animal data considered ‘reasonably likely’ to predict clinical benefit in humans.
